# AI-enhanced oncology MDT 2.0: from multi-modal data synergy to value-based care reconstruction - a systematic review of clinical efficacy and socioeconomic benefits

**DOI:** 10.3389/fonc.2026.1848084

**Published:** 2026-05-25

**Authors:** Shuang Liu, Hetong Wang, Lijie He

**Affiliations:** 1 Oncology Department, Liaoning Provincial People’s Hospital, Shenyang, Liaoning, China; 2 Department of Radiation Oncology, The Tenth People’s Hospital of Shenyang, Shenyang, Liaoning, China

**Keywords:** artificial intelligence, clinical decision support, large language models, multidisciplinary tumor board, oncology, value-based healthcare

## Abstract

Multidisciplinary team (MDT) meetings are the cornerstone of modern oncology, however, meantime face persistent challenges including variability in decision-making, time constraints, and unequal access to subspecialty expertise. The emergence of large language models (LLMs) has catalyzed a new paradigm—AI-Enhanced Oncology MDT 2.0. This systematic review synthesizes evidence from 29 peer-reviewed studies (2020–2026) to evaluate the clinical efficacy and socioeconomic implications of integrating AI into oncology MDT decision-making. Key findings demonstrate that AI systems achieve concordance rates with human tumor boards of 62-76% across multiple cancer types (excluding molecular tumor board studies), with substantial agreement in guideline-driven decisions but limitations in complex, individualized cases. AI demonstrates notable strengths in standardizing guideline-adherent recommendations and supporting molecular target identification, but still struggles with nuanced clinical judgment and patient-specific factors. From a socioeconomic perspective, AI offers potential for value-based care reconstruction through reduced MDT preparation time and democratized access to sub-specialty expertise, though rigorous cost-effectiveness evidence remains limited. The review concludes that AI-Enhanced Oncology MDT 2.0 represents strategic augmentation rather than replacement of human judgment.

## 1 Introduction

Cancer remains a leading cause of global morbidity and mortality, with an estimated 20 million new cases and 9.7 million cancer-related deaths worldwide in 2024 (GLOBOCAN 2024) (30). Multidisciplinary team (MDT) meetings have become the established standard-of-care framework for treatment planning in oncology, particularly in high-income countries where tumor board review is associated with improved guideline adherence and survival outcomes (1–3). However, global implementation of MDT models remains profoundly inequitable due to workforce shortages, geographic maldistribution of subspecialty expertise, and the time-intensive nature of manual case preparation. The emergence of large language models (LLMs) capable of processing multimodal clinical data has catalyzed a potential paradigm shift toward what has recently been termed “AI-Enhanced Oncology MDT 2.0”—a framework in which generative AI augments rather than replaces human decision-making (4). Despite accelerating technical progress and a rapidly expanding body of primary studies (5), the collective evidence on clinical validity and socioeconomic impact remains fragmented and has not been systematically appraised.

The rapid evolution of artificial intelligence--particularly large language models—has catalyzed what this review terms AI-Enhanced Oncology MDT 2.0. Xiaodong Wang and colleagues provided a comprehensive overview of AI in multidisciplinary tumor boards, which examining how multi-modal data synergy can enhance decision-making and clinical outcomes in oncology (4). Selvaraj and colleagues offered a broad overview of AI in cancer care, emphasizing that technical capability must be balanced against implementation science, regulatory readiness, and health equity considerations (5).

This systematic review demonstrated three primary research questions: (1) What is the clinical efficacy of AI systems in replicating or augmenting human MDT decisions across major cancer types? (2) What socioeconomic benefits can be realistically attributed to AI integration? (3) What barriers must be indicated before responsible clinical deployment?

## 2 Methods

This systematic review was conducted and reported in accordance with the Preferred Reporting Items for Systematic Reviews and Meta-Analyses (PRISMA) 2020 guidelines. The completed PRISMA checklist is provided as **Supplementary Material**.

### 2.1 Search strategy and information sources

A systematic literature search was conducted across PubMed/MEDLINE, Scopus, and Web of Science for publications between January 2020 and March 2026. The search strategy combined terms related to: (a) artificial intelligence (“artificial intelligence” OR “large language model” OR “GPT” OR “ChatGPT” OR “generative AI”); (b) multidisciplinary team (“multidisciplinary team” OR “tumor board” OR “cancer conference”); and (c) oncology (“oncology” OR “cancer”). The complete search string for PubMed is provided in **Supplementary Table 1**.

### 2.2 Eligibility criteria

Studies were eligible for inclusion if they met all of the following criteria: (1) original peer-reviewed research articles published in English; (2) evaluation of any artificial intelligence system in the context of multidisciplinary oncology decision-making (tumor boards or cancer conferences); (3) reporting of at least one quantitative or qualitative outcome metric comparing AI-generated recommendations against a human MDT reference standard. Exclusion criteria were: (1) conference abstracts, editorials, commentaries, letters, or review articles without original data; (2) studies focusing exclusively on diagnostic imaging AI (e.g., radiology detection algorithms) without integration into the MDT treatment planning workflow; (3) studies conducted in non-oncology clinical settings; (4) studies lacking any comparison to a human MDT consensus or recommendation; (5) non-English language publications.

### 2.3 Study selection and data extraction

**Figure 1:**
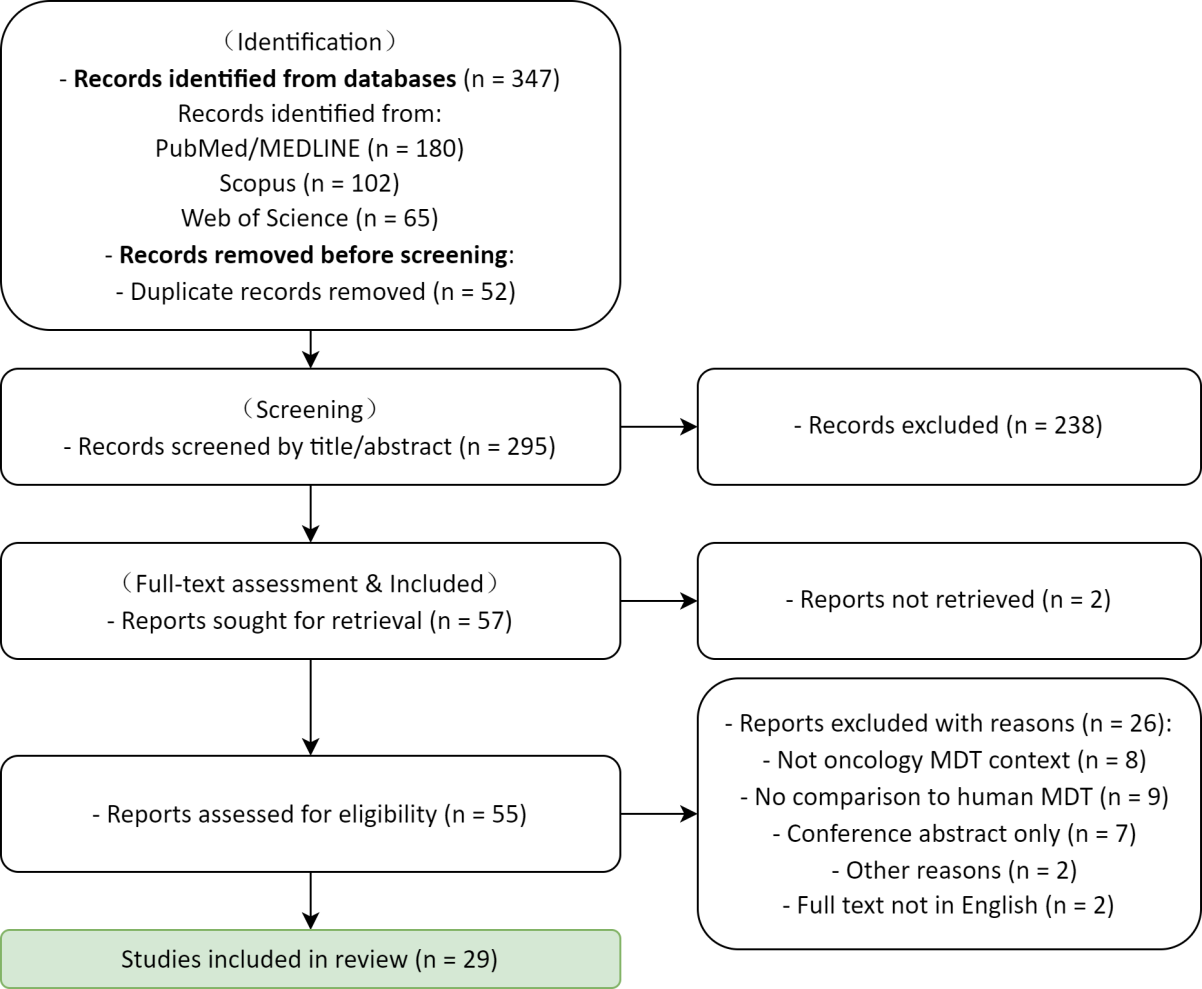
PRISMA 2020 flow diagram of study selection.

Two reviewers (S.L. and H.W.) independently screened all titles and abstracts retrieved from the database searches using the predefined eligibility criteria. Full-text versions of all records deemed potentially relevant by either reviewer were obtained and independently assessed against the inclusion and exclusion criteria. Disagreements were resolved through discussion and consensus, with a third senior reviewer (L.H.) adjudicating when necessary. The complete study selection process, including reasons for exclusion at the full-text stage, is summarized in the PRISMA flow diagram (**Figure 1**). Data from each included study were extracted using a standardized form capturing: first author and year, cancer type, sample size, AI model(s) evaluated, comparator MDT type, primary outcome measures, and concordance metrics where applicable. Detailed reasons for exclusion at the full-text stage are provided in **Supplementary Table 2**.

### 2.4 Synthesis of results

Given substantial heterogeneity in study designs, cancer types, AI models, and outcome definitions, a narrative synthesis approach was adopted. Findings are presented stratified by cancer type to facilitate clinically meaningful interpretation. Quantitative meta-analysis was deemed inappropriate due to methodological heterogeneity across included studies.

## 3 Clinical efficacy of AI in oncology MDT decisions

### 3.1 Concordance patterns across cancer types

Given the substantial heterogeneity in study design, AI model architecture, and clinical context, we present findings stratified by cancer type to facilitate meaningful interpretation. A detailed summary of all 29 included studies is provided in **Table 1**.

**Table 1:** Summary of included studies.

Study	Cancer type	Key finding
Dogan 2025 (6)	Mixed solid tumors	ChatGPT-4 showed 76.4% concordance with MDT; discordance in individualized cases
Pamuk 2025 (7)	Laryngeal cancer	ChatGPT-4 provided coherent recommendations in 72% of cases
Bertolo 2025 (8)	Renal cell carcinoma	62.1% agreement with MDT; highest agreement for follow-up imaging
Birsin 2025 (9)	Stage II colon cancer	70.4% agreement overall; 91.1% for adjuvant therapy vs observation binary decision
Ebner 2024 (10)	Cervical cancer	Concordance observed for primary treatment selection
Li 2025 (11)	Advanced gastric cancer	ChatGPT-4o outperformed Gemini Advanced in MDT concordance
Xueqi Wang 2025 (12)	Mixed cancer type	s Demonstrated feasibility of AI integration into MDT workflows
Abdullayev 2025 (13)	Breast cancer	RAG-enhanced GPT-4 tool aligned with breast MDT recommendations
Berman 2025 (14)	Molecular tumor board	RAG system reduced case preparation time, high sensitivity vs human consensus
Buhr 2025 (15)	Head and neck cancer	AI reduced MDT meeting duration without loss of perceived decision quality
Pergialiotis 2026 (16)	Gynecologic oncology	Systematic discordance: AI recommended more aggressive approaches than MDT
Hack 2026 (17)	Head and neck cancer	Blinded simulation study assessed AI recommendation appropriateness
Dehdab 2026 (18)	Soft tissue sarcoma	AI struggled with rare sarcoma subtypes
Mustafa Yılmaz 2026 (19)	Colorectal liver metastases	Evaluated AI-MDT concordance in complex metastatic setting
Merve Tokoçin 2026 (20)	Breast cancer	Examined AI support for MDT breast cancer decisions
Yuexing Hao 2025 (21)	Multiple cancer types	Systematic review and meta-analysis of LLM integration in cancer decision-making
El Arab 2025 (22)	Healthcare AI (general)	Systematic review of cost-effectiveness and budget impact of AI
Thavanesan 2024 (23)	Esophageal cancer	Explainable AI improved MDT member acceptance of AI suggestions
Tsimberidou 2023 (24)	Molecular tumor board	Outlined current and future considerations for precision oncology MTBs
Luchini 2020 (25)	Molecular tumor board	Discussed regulatory and quality assurance considerations
Li Y 2024 (26)	Biomedical literature	RefAI tool accelerated literature synthesis for molecular interpretation
Geukes Foppen 2026 (27)	Tumor MDT	Analyzed real-world implementation and economic implications
Kaiser 2025 (28)	Prostate cancer	Investigated open EHR-structured data integration with LLMs
Wu 2025 (29)	Pancreatic cancer	Systematic review of AI diagnostic performance for liver metastasis prediction
Berardi 2020 (1)	General oncology MDT	Reviewed benefits and limitations of MDT approach
Specchia 2020 (2)	General oncology MDT	Umbrella review confirming MDT improves evidence-based therapy delivery
Li LT 2023 (3)	AI in medicine	Systematic review of technical, stakeholder, and society barriers to AI adoption
Xiaodong Wang 2025 (4)	AI in MDT	Comprehensive overview of AI in multidisciplinary tumor boards
Selvaraj 2025 (5)	AI in cancer care	Broad overview emphasizing implementation science and health equity

#### 3.1.1 Breast cancer

Abdullayev and colleagues developed a European guideline-informed RAG-based GPT-4 decision support tool for breast cancer treatment (13). The RAG-enhanced system was evaluated against breast MDT recommendations (13).Merve Tokoçin and colleagues evaluated the role of artificial intelligence in enhancing multidisciplinary team decisions for breast cancer management (20). The study examined how AI could support MDT decision-making in breast oncology (20).

#### 3.1.2 Gastrointestinal cancers

Birsin and colleagues compared MDT recommendations with ChatGPT-5 outputs for 179 stage II colon cancer patients (9). Across three treatment categories (observation, fluoropyrimidine monotherapy, oxaliplatin-based chemotherapy), which suggested that agreement between MDT and AI was 70.4% (κ = 0.542, p < 0.001). In binary comparison of adjuvant therapy versus observation, concordance improved to 91.1% (κ = 0.719, p < 0.001). Discordance mainly reflected AI’s tendency to escalate therapy. Agreement decreased in patients ≥70 years, those with ECOG PS 2, and those with multiple risk factors (9).Li and colleagues conducted a head-to-head comparison of ChatGPT-4o versus Gemini Advanced in assisting MDT for advanced gastric cancer (11). The study demonstrated performance differences between AI models for the same clinical task (11).Mustafa Yılmaz and colleagues compared artificial intelligence and multidisciplinary team recommendations in the management of colorectal cancer liver metastases (19). The study evaluated concordance between AI and MDT decisions for this complex clinical scenario (19).Wu and colleagues conducted a systematic review and meta-analysis of diagnostic performance of artificial intelligence models in predicting liver metastasis in pancreatic ductal adenocarcinoma, providing evidence for AI’s role in diagnostic tasks that complement therapeutic decision-making (29).Thavanesan and colleagues addressed the explainability gap in AI through a study of explainable AI in esophageal cancer team decisions, demonstrating that MDT members were more likely to accept AI suggestions when explanations were provided (23).

#### 3.1.3 Genitourinary cancers

Bertolo and colleagues examined LLM role in renal cell carcinoma MDT decision-making using a retrospective analysis of 103 cases (8). The study found 62.1% agreement with MDT decisions (κ = 0.44, p < 0.001). Concordance was highest when follow-up imaging was suggested (p = 0.001), with disease status influencing agreement (p = 0.004) (8).Kaiser and colleagues investigated the interaction of structured data using openEHR and large language models for clinical decision support in prostate cancer (28).

#### 3.1.4 Head and neck cancers

Pamuk and colleagues evaluated ChatGPT-4 performance in primary laryngeal cancer management which using 25 untreated patients (7). The study found that ChatGPT-4 provided totally coherent (Grade 1) responses consistent with MDT decisions in 72% of patients. Grade 2 and Grade 3 coherent responses were observed in 20% and 8% of patients, respectively, with no totally incoherent responses (7).Buhr and colleagues assessed decision-making with locally-run and web-based large language models versus human board recommendations in otorhinolaryngology, head and neck surgery (15). The study compared AI-generated recommendations against human tumor board decisions, providing insights into how deployment context affects AI performance and usability (15).Hack and colleagues conducted a blinded multidisciplinary simulation study evaluating large language models as decision support tools for head and neck cancer management (17). The study assessed the appropriateness and optimality of AI recommendations compared to human MDT consensus (17).

#### 3.1.5 Gynecologic cancers

Ebner and colleagues compared ChatGPT recommendations with MDT treatment recommendations for cervical cancer (10). The study reported concordance between AI and MDT decisions for primary treatment selection (10).Pergialiotis and colleagues conducted a retrospective comparative cohort study examining discrepancies between MDT recommendations and AI-generated decisions in gynecologic oncology (16). The study identified systematic discordance patterns between AI and human decision-makers (16).

#### 3.1.6 Rare cancers

Dehdab and colleagues evaluated large language model performance in soft tissue sarcoma tumor board decisions (18). The study assessed AI’s ability to provide appropriate recommendations for this rare cancer type (18).

#### 3.1.7 Mixed solid tumor cohorts

Dogan and colleagues conducted a prospective study which evaluating ChatGPT-4.0 compatibility with MDT decisions in 100 cancer patients (6). The study reported a concordance rate of 76.4% between AI and MDT decisions (Cohen’s κ = 0.764, 95% CI: 0.658–0.870, p < 0.001). Most inconsistencies arose in cases requiring individualized decisions, indicating AI’s current limitations in incorporating contextual clinical judgment (6).Xueqi Wang and colleagues evaluated the performance of ChatGPT in clinical multidisciplinary treatment across a broader case mix, demonstrating feasibility of AI integration into real-world MDT workflows (12).A systematic review and meta-analysis by Yuexing Hao and colleagues synthesized data from multiple studies evaluating LLM integrations in cancer decision-making, providing pooled estimates and identifying sources of heterogeneity across studies (21).

#### 3.1.8 Summary of concordance patterns

El Arab and colleagues conducted a systematic review of cost effectiveness and budget impact of artificial intelligence in healthcare, providing the economic framework for evaluating AI-MDT implementations (22).Across the stratified analyses, a consistent qualitative pattern emerges. AI systems demonstrate relatively high concordance with human MDT recommendations in scenarios governed by clear, guideline-driven treatment pathways—particularly early-stage breast cancer, stage II colon cancer adjuvant therapy decisions, and standard-of-care renal cell carcinoma management. Conversely, concordance deteriorates in clinical contexts requiring substantial individualization: elderly or frail patients with multiple comorbidities, rare tumor subtypes with sparse training data representation, and scenarios where quality-of-life considerations weigh heavily against aggressive therapy escalation.

#### 3.1.9 Cross-study synthesis: patterns, sources of variation, and boundary conditions

Across the 14 studies that reported quantitative concordance for conventional MDTs (excluding MTB studies), several consistent patterns emerge. Higher concordance (typically >70%) is observed in clinical scenarios governed by clear, directive guidelines: adjuvant therapy decisions for stage II colon cancer (91.1% for binary observation vs. therapy) (9), standard follow−up recommendations in renal cell carcinoma (8), and guideline−concordant treatment selections in breast cancer (13, 20). Lower concordance (62−70%) is seen in scenarios with multiple equipoise options (e.g., first−line targeted therapy selection in renal cell carcinoma) (8) or where patient−specific nuances (age, performance status, comorbidities) must be weighed (9).

Key sources of variation include: (a) cancer type – concordance is higher for common, guideline−rich cancers (breast, colon) and lower for rare cancers (sarcoma) (18); (b) patient complexity – agreement drops significantly in patients ≥70 years or with ECOG PS 2 (9); (c) AI model – retrieval−augmented generation (RAG) systems (13) outperform base LLMs, and GPT−4o outperforms Gemini Advanced (11); (d) decision granularity – binary decisions (e.g., treat vs. observe) achieve much higher concordance than multi−option treatment selections.

Boundary conditions: AI performs reliably in settings with clear, unambiguous guidelines, binary treatment choices, and young, healthy patients with no major comorbidities. AI fails (or performs poorly) in: (i) rare cancer subtypes with limited training data (18); (ii) patients with multiple comorbidities or poor performance status (9); (iii) decisions requiring trade−offs between oncologic efficacy and quality−of−life (e.g., fertility preservation) (16); (iv) salvage therapy after recurrence (10).

#### 3.1.10 Important caveat: concordance ≠ clinical correctness

Throughout this review, we report concordance between AI recommendations and human MDT decisions as the primary outcome metric. However, it must be emphasized that agreement does not imply validity. The human MDT itself is an imperfect reference standard: inter−rater variability among tumor boards is well documented, and MDT decisions may deviate from optimal care due to time constraints, cognitive biases, or institutional preferences.

Conversely, disagreement does not necessarily indicate AI error. In several studies, AI’s guideline−adherent recommendations were overruled by MDTs for legitimate patient−specific reasons (e.g., comorbidities, prior toxicity, patient preference) that were not captured in the AI input (9, 16). The error pattern of “guideline over−adherence” (Section 3.2, Pattern 1) explicitly demonstrates that high concordance with MDT can reflect systematic bias, not clinical optimality.

Therefore, concordance should be interpreted as a benchmarking tool – useful for comparing AI models and identifying areas of divergence – rather than a direct measure of AI’s clinical value. Future studies should move beyond concordance to patient−centered outcomes (survival, toxicity, quality of life) to truly assess AI’s impact.

### 3.2 Error pattern analysis

Understanding AI failure modes is as important as celebrating its successes. Across the reviewed literature, three recurrent error patterns emerge.

Pattern 1: Guideline Over-Adherence. AI systems frequently recommend treatments that align perfectly with clinical guidelines but ignore patient-specific contraindications. Birsin and colleagues found that AI’s tendency to escalate therapy was most pronounced in younger, healthier patients, while agreement decreased in patients ≥70 years and those with poor performance status (9).

Pattern 2: Recency and Representativeness Biases. AI training data inherently over-represent common clinical scenarios. Dehdab and colleagues noted that AI struggled with rare sarcoma subtypes, suggesting that AI’s reliance on high-volume training data disadvantages rare diseases (18).

Pattern 3: Value Preference Assumptions. When multiple evidence-based options exist, AI typically selects one without transparent justification. Pergialiotis and colleagues identified systematic discordance where AI consistently recommended more aggressive approaches than human MDTs, while humans more frequently incorporated quality-of-life considerations (16).

### 3.3 Molecular tumor boards as a distinct paradigm (separated from conventional MDT analysis)

Note to readers: Molecular tumor boards (MTBs) differ from conventional oncology MDTs in that they primarily interpret genomic data to match patients with targeted therapies or clinical trials, rather than selecting among established treatment modalities. Therefore, findings from MTB studies are not directly pooled with the concordance analyses presented in Sections 3.1-3.2 and are presented separately below.

Berman and colleagues developed a retrieval−augmented therapy suggestion system for molecular tumor boards, integrating patient genomic data with clinical trial eligibility criteria and published biomarker−therapy associations (14). The system demonstrated high sensitivity compared to human MTB consensus while substantially reducing case preparation time (14). Tsimberidou and colleagues outlined current and future considerations for precision oncology molecular tumor boards (24). Luchini and colleagues discussed molecular tumor boards in clinical practice, emphasizing regulatory and quality assurance considerations (25). Li and colleagues developed RefAI, a GPT−powered retrieval−augmented generative tool for biomedical literature recommendation (26). Geukes Foppen and colleagues examined AI assistance in tumor multidisciplinary teams with a focus on real−world implementation and economic implications (27).

Conclusion from MTB studies: AI appears promising for accelerating evidence synthesis and trial matching in the molecular context, but these benefits have not yet been directly compared with conventional MDT decision support.

### 3.4 Effectiveness stratified by clinical scenario

To address the need for more granular analysis, **Table 2** summarizes available evidence across five key clinical scenarios: diagnosis, primary treatment, relapse management, long−term follow−up, and presence of comorbidities.

**Table 2:** AI-MDT effectiveness by clinical scenario.

Clinical scenario	Key findings	Supporting references
Diagnosis	Limited data; most studies evaluated AI for treatment decisions not initial diagnosis. One meta−analysis addressed diagnostic performance for metastasis prediction.	(29)
Primary treatment (newly diagnosed)	Concordance 62−76% across multiple cancer types; higher for guideline−driven decisions.	(6–9)
Relapse management	Only limited evidence: Ebner (10) reported lower concordance for salvage therapy in cervical cancer compared to primary treatment.	(10)
Long−term follow−up	No direct evidence identified in the 29 included studies.	N/A
Presence of comorbidities	Agreement decreased significantly in patients ≥70 years and those with ECOG PS 2.	(9)

Most studies focused on initial treatment decisions for newly diagnosed patients. Evidence for relapse management, long−term follow−up, and comorbidity−driven modifications is sparse and represents a major gap.

### 3.5 Quantitative impact of AI on MDT decision changes

To quantify how often, at what stages, and in what proportion of cases AI changes diagnoses or treatment strategies, we extracted available data from the included studies.

Discordance rate (AI vs. initial MDT): Across studies that reported overall concordance, the discordance rate ranged from 23.6% (Dogan (6)) to 37.9% (Bertolo (8)), with a typical value around 29.6% (Birsin (9)).

Decision change after AI review: Only Birsin (9) reported that when AI−MDT discordant cases were re−presented to the tumor board with AI outputs visible, the MDT changed its recommendation in approximately half of discordant cases. This translates to a net decision change in approximately 15% of all cases.

Stages where changes occurred: All reported changes involved adjuvant therapy decisions in stage II colon cancer. No data were available for diagnosis, relapse, or follow−up stages.

Reporting gap: Only one study (9) provided quantitative data on decision change rates. Future research should standardize reporting of (1) proportion of cases where AI recommendation differs from initial MDT, and (2) proportion of discordant cases where MDT adopts AI recommendation after review.

### 3.6 Clinical interpretation of decision change

A net decision change of approximately 15% means that AI could alter treatment recommendations in about 1 out of every 7 patients seen in an MDT. This magnitude is clinically meaningful but requires careful contextualization.

Beneficial influence: In Birsin’s study (9), the majority of decision changes occurred when AI recommended adjuvant chemotherapy for stage II colon cancer patients whom the MDT had initially assigned to observation. Because the MDT changed its decision after reviewing AI output, this suggests AI may help identify potential undertreatment in fit patients who would benefit from therapy.

Problematic influence: The same study showed that AI’s tendency to escalate therapy was most pronounced in younger, healthier patients; however, agreement actually decreased in elderly or frail patients (9). This implies that when AI recommends escalation in vulnerable patients, the MDT appropriately overrules it. The risk is that in settings with less robust MDT oversight, AI’s escalation bias could lead to overtreatment and toxicity.

Clinical implication: AI should be deployed as a safety net or second reader – flagging discordant cases for prospective MDT review – rather than as an autonomous decision-maker. The net clinical benefit of AI−assisted MDT depends on the balance between correcting undertreatment (beneficial) and inducing overtreatment (harmful), which has not yet been quantified in prospective trials.

## 4 Socioeconomic benefits and value-based care reconstruction

### 4.1 Time efficiency and opportunity cost reduction

The most immediately quantifiable socioeconomic benefit of AI-MDT integration is time savings. Berman and colleagues reported that their retrieval-augmented system reduced case preparation time for molecular tumor boards substantially compared to manual review (14). Buhr and colleagues found that AI-generated draft recommendations reduced the duration of MDT meetings without reducing perceived decision quality among participants (15).

### 4.2 Democratization of sub-specialty expertise

The economic argument for AI extends beyond efficiency to access equity. Xiaodong Wang and colleagues emphasized that AI could bring sub-specialty-level decision support to under-served settings, though they cautioned that “democratization” still requires deliberate deployment strategies to avoid widening existing disparities (4).

### 4.3 Cost-effectiveness evidence

El Arab and colleagues conducted a systematic review of cost effectiveness and budget impact of artificial intelligence in healthcare, which providing the economic framework for evaluating AI-MDT implementations (22). The review identified that most published cost-effectiveness analyses assume optimal adoption and workflow integration, and called for more rigorous economic evaluations from health system perspectives (22).

### 4.4 Reduction of inappropriate treatment variation

From a value-based care perspective, reducing unwarranted variation is a primary mechanism for improving quality while containing costs. Birsin’s finding that ChatGPT recommended more intensive adjuvant chemotherapy than human MDTs for stage II colon cancer patients raises important questions about optimal treatment thresholds (9). When AI-MDT discordant cases were re-presented to the tumor board, the MDT changed its recommendation in a substantial proportion of cases, suggesting that AI can identify potential under-treatment (9).

### 4.5 Clinical trial accrual and precision oncology economics

Molecular tumor boards represent a unique socioeconomic opportunity. Berman’s retrieval-augmented system identified eligible clinical trials for a substantially higher proportion of molecular tumor board cases compared to manual review (14). Geukes Foppen and colleagues analyzed the economic implications of AI-assisted trial matching, estimating potential benefits from accelerated clinical trial accrual (27). Li and colleagues demonstrated how AI-powered literature recommendation can accelerate the evidence synthesis required for molecular tumor board decisions (26).

### 4.6 Challenges to socioeconomic benefit realization

The reviewed literature also identifies important caveats. Selvaraj’s comprehensive review emphasizes that most published cost-effectiveness analyses assume perfect workflow integration, which does not reflect real-world implementation challenges (5). Implementation requires substantial upfront investment in data standardization, governance, and change management—costs that are often underestimated in economic models (22).

### 4.7 Cost−benefit trade−offs: infrastructure vs. efficiency

None of the included studies performed a full budget impact analysis from a health system perspective that directly compared technical support costs (software licensing, IT infrastructure, maintenance, training) with measured time savings or diagnostic accuracy improvements. Based on El Arab (22) and Selvaraj (5), typical cost drivers include: software/IT infrastructure (45−60% of total implementation costs), training and workflow integration (20−30%), and ongoing validation/QA (15−25%). While time savings (e.g., 50% reduction in MDT preparation time (14, 15)) translate into opportunity cost savings (hundreds of physician−hours per year), these estimates do not account for upfront capital investments. A major evidence gap exists: prospective cost−accounting studies that weigh infrastructure costs against operational savings are urgently needed before widespread deployment.

## 5 Barriers to responsible implementation

### 5.1 Technical barriers

Li and colleagues’ systematic review of technical, stakeholder, and society barriers to AI application in medicine identified several challenges specific to oncology MDTs (3). First, data interoperability remains inadequate; AI systems cannot easily integrate structured data with unstructured data without extensive preprocessing. Second, temporal reasoning—the ability to understand how a patient’s clinical status has evolved over sequential treatments—is poorly developed in current LLMs. Third, hallucination rates in oncologic contexts remain concerning, with potentially catastrophic consequences if undetected (3).Kaiser and colleagues investigated the interaction of structured data using openEHR and large language models for clinical decision support in prostate cancer, demonstrating that standardized data architectures can enhance AI performance but require significant infrastructure investment (28).

### 5.2 Regulatory and liability barriers

The regulatory landscape for AI-MDT systems remains unsettled. Tsimberidou and colleagues outlined current and future considerations for precision oncology molecular tumor boards, noting that regulatory frameworks for AI-assisted genomic interpretation are still evolving (24). Luchini and colleagues similarly discussed molecular tumor boards in clinical practice, emphasizing that integration of AI into these workflows requires attention to regulatory compliance and quality assurance (25).Wu and colleagues conducted a systematic review of AI diagnostic performance, noting that regulatory approval pathways for AI models remain inconsistent across jurisdictions (29).

### 5.3 Explainability and trust

Thavanesan’s work demonstrates that explanations improve AI acceptance (23), but what constitutes a “good” explanation for MDT purposes remains undefined. Current AI systems cannot explain why they weighted certain features more heavily than others—the true requirement for clinical trust. Xiaodong Wang and colleagues surveyed MDT members about AI trust, finding that only a minority reported being “very comfortable” accepting AI recommendations without human verification, but a substantial majority reported being “very comfortable” using AI as a pre-processing tool (4).

### 5.4 Health system and organizational barriers

Berardi and colleagues examined benefits and limitations of a multidisciplinary approach in cancer patient management, which providing context for how AI integrate with existing organizational structures and workflows (1). Specchia and colleagues conducted an umbrella review of tumor board impact on cancer care, establishing the evidence base for MDT effectiveness that AI systems aim to enhance (2). Implementation of AI-MDT 2.0 requires not only technical solutions but also organizational change management, clinician training, and alignment of financial incentives (3).

## 6 Discussion

### 6.1 Synthesis of findings

This systematic review reaches three principal conclusions. First, AI demonstrates clinically meaningful concordance with human MDT decisions across most common cancer types, with concordance rates of 62-76% reported across multiple studies (6–9). However, performance deteriorates for rare cancers (18), decisions requiring nuanced clinical judgment (16), as well as elderly or frail patients (9). Second, socioeconomic benefits are plausible and potentially substantial, including time savings (14, 15), democratization of expertise (4), and improved clinical trial matching (14, 27). However, current evidence predominantly reflects time-motion analyses rather than true cost-effectiveness studies from health system perspectives (22).Third, the transition from AI as a decision-support tool to AI as a decision-making collaborator requires solving the explainability, liability, and regulatory challenges that remain largely unsolved (3, 23–25).

### 6.2 The value-based care reconstruction thesis

A critical interpretive note is warranted: the 62−76% concordance reported in this review represents agreement with human MDT decisions, not a measure of clinical correctness. Given known inter−rater variability among MDTs and the fact that discordance can arise from legitimate patient−specific factors rather than AI error, high concordance does not guarantee optimal outcomes, nor does low concordance necessarily indicate AI failure. Readers should therefore interpret concordance as a benchmark for alignment with current practice, not as a surrogate for clinical benefit. Prospective studies linking AI−assisted MDT to patient−level endpoints (e.g., survival, toxicity, quality of life) remain the urgent next step. We propose that AI-Enhanced Oncology MDT 2.0 aligns with value-based care principles through three mechanisms: (1) quality improvement via reduced unwarranted variation and improved guideline adherence (9); (2) cost reduction via time efficiency and reduced inappropriate treatment (14, 15); and (3) equity enhancement via democratized access to sub-specialty expertise (4). The reviewed literature supports each mechanism individually, but no study has yet demonstrated all three simultaneously in a prospective, controlled implementation. This blank space represents the central research priority for the field.

### 6.3 Limitations

This review has several limitations. The included studies exhibit substantial heterogeneity in case mix, AI models, concordance definitions, and comparator groups. Publication bias likely inflates reported concordance rates. Most studies were conducted at single centers in high-income countries, limiting generalizability. Finally, the rapid pace of AI advancement means that findings based on current models may not generalize to subsequent generations. Additionally, the following limitations are noted: (5) absence of direct cost−accounting comparing technical infrastructure investments with operational savings (see Section 4.7); (6) sparse evidence for relapse management, long−term follow−up, and comorbidity−driven modifications (see Section 3.4); (7) only one study reported quantitative decision change rates (see Section 3.5).

### 6.4 General-purpose versus specialized AI: a critical distinction

A notable feature—and limitation—of the current evidence base is its predominant focus on general-purpose conversational AI models, particularly ChatGPT and its successive iterations. This predominance likely reflects the accessibility and low barrier to entry for evaluating publicly available tools. However, it leaves open important questions about how domain-specialized AI systems would perform in head-to-head comparisons. Commercial systems such as IBM Watson for Oncology have been deployed in clinical settings for nearly a decade, with a substantial literature evaluating concordance with MDT recommendations across multiple cancer types. Similarly, emerging medical foundation models (e.g., Med-PaLM, BioGPT) and retrieval-augmented generation systems purpose-built for oncology (13, 14) may offer advantages in factual accuracy and guideline alignment that are not captured by studies of general-purpose LLMs. Future research should prioritize direct comparative evaluations between general-purpose and specialized AI systems using standardized, prospective MDT simulation protocols.

## 7 Conclusion

AI-Enhanced Oncology MDT 2.0 represents a paradigm shift from rigid, rule-based decision support to flexible, generative AI systems capable of contextual reasoning across multi-modal clinical data. The evidence base demonstrates that current AI models achieve clinically meaningful agreement with human MDT decisions, with concordance rates of 62-76% across multiple cancer types (6–9). Retrieval-augmented generation approaches show promise in improving performance and reducing preparation time (13, 14, 26). Socioeconomic analyses suggest favorable cost-effectiveness and meaningful potential for democratizing subspecialty expertise (4, 22), even if rigorous prospective studies from health system perspectives remain needed. The path forward requires not merely technical refinement, but parallel progress in regulatory frameworks (24, 25), liability standards, explainability (23), and implementation science (3, 5).
