# Supplementary material for: AI-enhanced oncology MDT 2.0: from multi-modal data synergy to value-based care reconstruction - a systematic review of clinical efficacy and socioeconomic benefits
Source: Front Oncol. 2026 May 25;16:1848084. doi: 10.3389/fonc.2026.1848084 (PMC13243095; doi:10.3389/fonc.2026.1848084)
Supplement: Supplementary file 1 [file SupplementaryFile1.docx]

Supplementary Table S1. PubMed Search Strategy

| Search | Query |
| --- | --- |
| #1 | "artificial intelligence"[Title/Abstract] OR "large language model"[Title/Abstract] OR "GPT"[Title/Abstract] OR "ChatGPT"[Title/Abstract] OR "generative AI"[Title/Abstract] |
| #2 | "multidisciplinary team"[Title/Abstract] OR "tumor board"[Title/Abstract] OR "cancer conference"[Title/Abstract] |
| #3 | "oncology"[Title/Abstract] OR "cancer"[Title/Abstract] |
| #4 | #1 AND #2 AND #3 |
| Filters | English Publication date from 2020/01/01 to 2026/03/31 |
