# Supplementary material for: AI-enhanced oncology MDT 2.0: from multi-modal data synergy to value-based care reconstruction - a systematic review of clinical efficacy and socioeconomic benefits
Source: Front Oncol. 2026 May 25;16:1848084. doi: 10.3389/fonc.2026.1848084 (PMC13243095; doi:10.3389/fonc.2026.1848084)
Supplement: Supplementary file 2 [file SupplementaryFile2.docx]

Supplementary Table S2. Detailed reasons for exclusion at the full-text review stage.

| Exclusion Reason (Exclusion Reason) | Number of Records (Number of Records) | Specific Definition (Specific Definition) |
| --- | --- | --- |
| Not oncology MDT context | 8 | Study did not evaluate AI in a tumor board or multidisciplinary cancer conference setting. |
| No comparison to human MDT | 9 | AI-generated recommendations were not directly compared against a human MDT consensus or reference standard. |
| Conference abstract only | 7 | Full peer-reviewed article was not available; only a conference abstract was published. |
| Full text not in English | 2 | The complete publication was in a language other than English, which was an exclusion criterion (Section 2.2). |
| Total Excluded | 26 |  |
